# Supplementary material for: Smoothelin-Like Protein 1 Regulates the Thyroid Hormone-Induced Homeostasis and Remodeling of C2C12 Cells via the Modulation of Myosin Phosphatase
Source: Int J Mol Sci. 2021 Sep 24;22(19):10293. doi: 10.3390/ijms221910293 (PMC8508602; doi:10.3390/ijms221910293)
Supplement: Supplementary file 1 [file ijms-22-10293-s001.zip › ijms-1361337-supplementary.pdf]

Supplementary Materials

Smoothelin-like protein 1 Regulates the Thyroid Hormone-Induced Homeostasis and Remodeling of C2C12 Cells via the Modulation of Myosin Phosphatase

Evelin Major[1], Ilka Keller[1], Dániel Horváth[1], István Tamás[1], Ferenc Erdődi[1], Beáta Lontay[1*]

[1]Department of Medical Chemistry, Faculty of Medicine, University of Debrecen

***Correspondence to:**

Dr. Beáta Lontay

Department of Medical Chemistry, Faculty of Medicine

University of Debrecen,

H-4032 Debrecen, Egyetem tér 1. Hungary

Phone: +36-52-412345

e-mail: lontay@med.unideb.hu

Running title: myosin phosphatase 1 regulation in hyperthyroidism

Supplementary Methods

Membrane Stripping with Heat and Detergent. Membranes were washed 3 times for 5 minutes with 1x TBST and incubated in Stripping buffer [0.5 M Tris-HCl pH 6.8; 10% SDS, β-mercaptoethanol] at 50 °C for 30 minutes in a Thermo-Shaker (Biosan Laboratories Inc., Warren, MI, USA). Next, membranes were washed 6 times for 5 minutes with 1x TBST and reblocked with 5% BSA/TBST at RT for 1 hour. Incubation with primary antibodies followed by secondary antibodies and visualization of immunoreactions were performed as described in the *Western Blot Analysis* section.

Horse Radish Peroxidase (HRP) Inactivation. This method was developed by Sennepin and coworkers [1] and involves a 15-min incubation of Western blot membranes with 1 ml of 30% (w/v) H_2O_2 solution at 37 °C without shaking. Then membranes are washed and reblocked with 5% BSA/ TBST at RT for 1 hour. Incubation with primary antibodies followed by secondary antibodies and visualization of immunoreactions were carried out as described in the *Western Blot Analysis* section.

Morphological Analysis. Myoblasts were transfected with empty vector or NT-FT-SMTNL1 and were differentiated for 5 days. On days 0, 3 and 5 of differentiation, light microscopy images of developing monolayers were taken with a 10x air objective and morphology analysis was performed by ImageJ software. The average area and perimeter of 15 randomly selected multinucleated myotubes per field were calculated in each case (n=3). All values were normalized to the average value of the control (Day 0 of the empty vector-transfected control), due to single cells were the major components here. The total number of myotubes present in a field was also counted.

Immunoprecipitation. Antibodies were coupled to protein A Sepharose (PAS) resin (GE Healthcare, Chicago, IL, USA, #17-0780-01) in the presence of binding buffer (50 mM Tris, pH 7.0). Beads then were blocked with 5% (w/v) BSA/TBST for 2 hours at 4 °C. Bead-antibody

complexes were washed three times with binding buffer; between each washing step Eppendorf-tubes were centrifuged at 800 x *g* at 4 °C for 3 min, then the supernatants were discarded. The bead-antibody complexes were incubated with the precleared (previously incubated with PAS) C2C12 lysates at 4 °C O/N. Finally, beads were washed three times with 50 mM Tris (pH 7.0), and the supernatants were completely eliminated using a Hamilton-pipette. 30 µl of 1x SDS sample buffer was added to the beads and samples were boiled at 100 °C for 5 min. Precipitates were subjected to Western blot.

Supplementary Figures

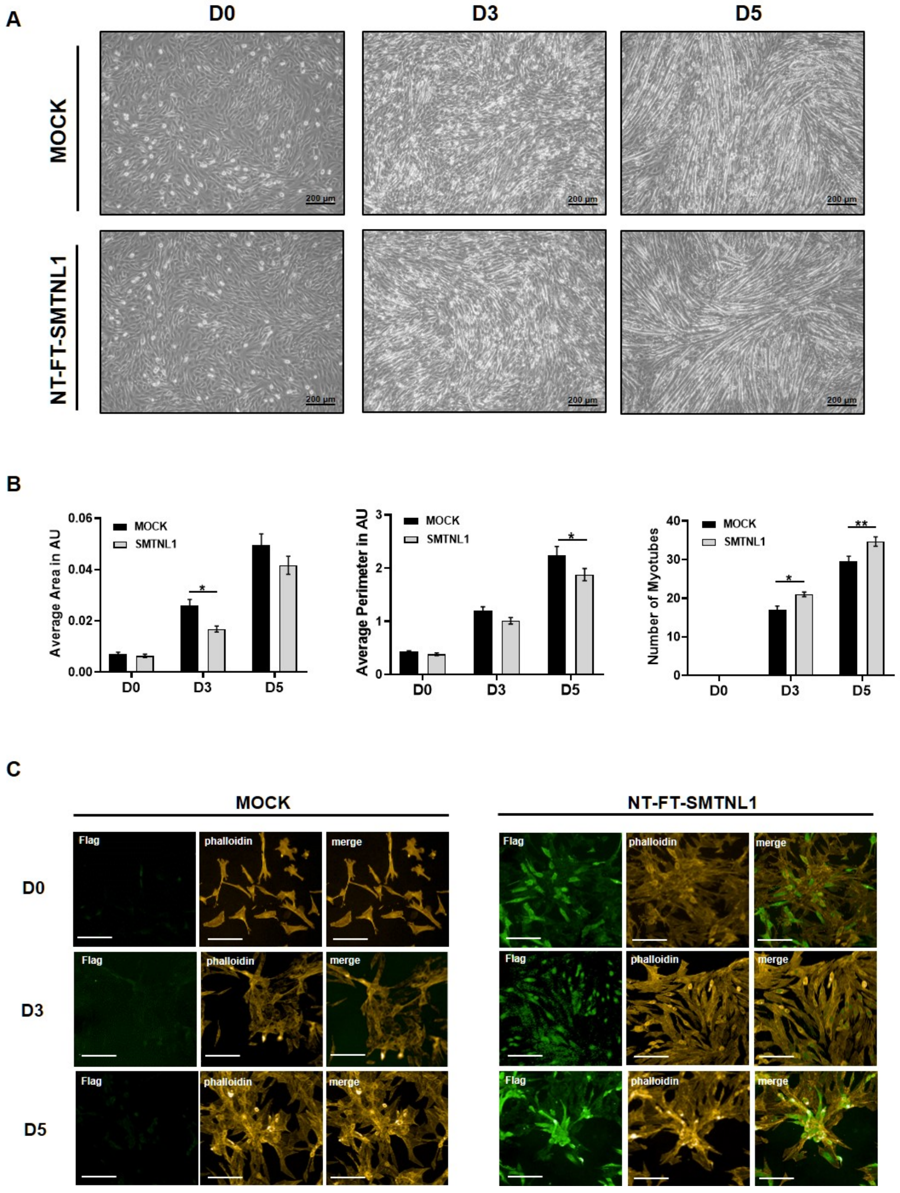

Figure S1. *Morphological Changes in Differentiated C2C12 Myotubes as a Result of SMTNL1 Overexpression* Light microscopy images were taken and used to calculate the average area, average perimeter and total myotube number by ImageJ software **(A-B)**. Scale bars represent: 200 µm. Values represent n=3, mean +/- SEM. Data were normalized to the Day 0 of empty vector-transfected control. Differences between group means were determined by Two-way ANOVA followed by Sidak's multiple comparisons *post hoc* test, $p < 0.05$ (*), $p < 0.01$ (**). **(C)** Immunofluorescence staining was performed on empty vector-transfected or NT-FT-SMTNL1-transfected C2C12 cells at D0, D3 and D5 of differentiation using anti-Flag antibody and Texas-Red phalloidin. Scale bars represent: 500 µm.

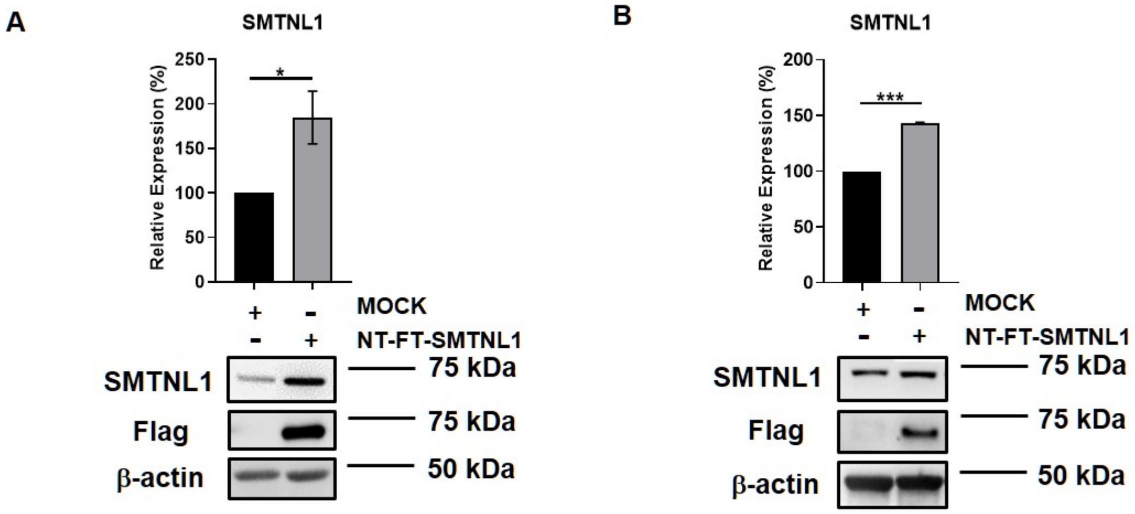

Figure S2. *Overexpression of Recombinant SMTNL1 Protein in C2C12 Myoblasts and Myotubes* Empty vector or NT-FT-SMTNL1-transfected myoblasts **(A)** and myotubes **(B)** were lysed and subjected to Western blot analysis. Overexpression was confirmed by using anti-Flag and anti-SMTNL1 antibodies **(A-B)**. Values represent n=4, mean +/- SEM. Data were normalized to the empty vector-transfected control. Differences between group means were determined by unpaired two-tailed t-test, $p < 0.05$ (*), $p < 0.001$ (***).

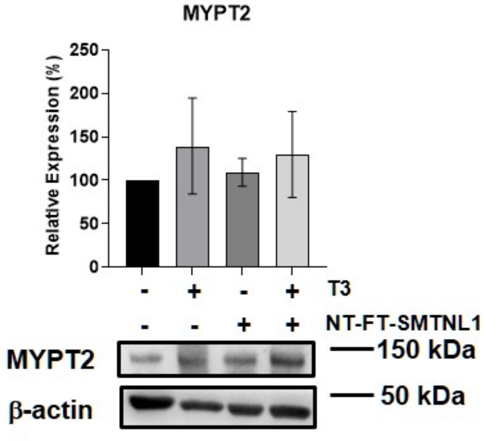

Figure S3. *T3 Treatment Regulates MYPT2 Expression in Myoblasts* NT-FT-SMTNL1-transfected myoblasts were treated with 10 nM T_3 for 24 hours. Whole-cell lysates were analyzed by Western blot using anti-MYPT2 antibody. Values represent n=3, mean +/- SEM. Data were normalized to the empty vector-transfected control. Differences between group means were determined by One-way ANOVA and Tukey's *post hoc* test, $p < 0.05$.

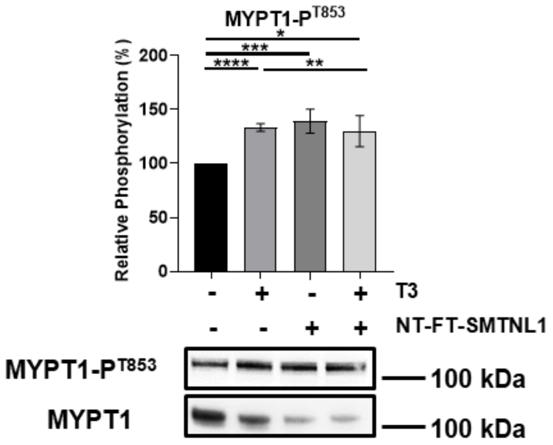

Figure S4. *The Effect of SMTNL1 Overexpression and T_3 Treatment on the Phosphorylation of MYPT1 in Myotubes* Myoblasts were transfected with empty vector or NT-FT-SMTNL1 and were differentiated for 6 days with a simultaneous 72-hour T_3 treatment started from Day 4. Proteins from whole cell lysates were analyzed by Western blot using anti-MYPT1-P^{T853} antibody. Values represent n=6, mean +/- SEM. Data were normalized to the empty vector-transfected control. Differences between group means were determined by One-way ANOVA and Tukey's *post hoc* test, p <0.05 (*), p <0.01 (**), p <0.001 (***) and p <0.0001 (****).

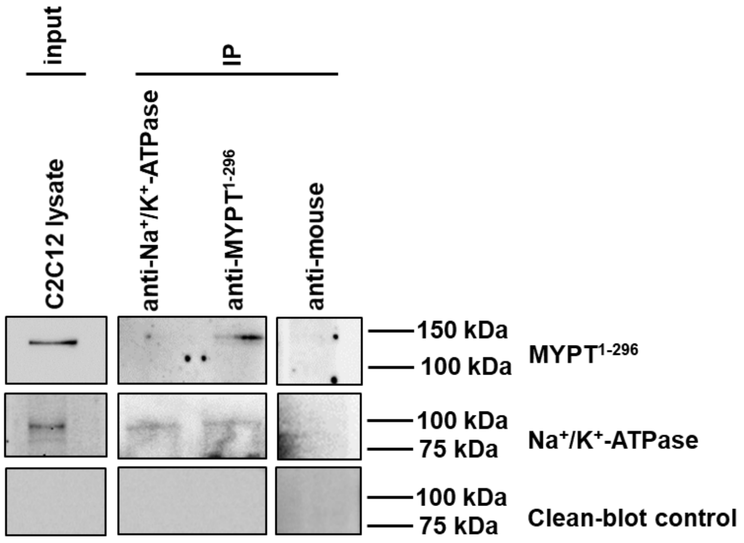

Figure S5. *Interaction between the Na$^+$/K$^+$-ATPase and MYPT in Myoblasts* Immunoprecipitation was performed on C2C12 lysates using anti-Na$^+$/K$^+$-ATPase, anti-

MYPT$^{1-296}$ and – as a negative control – anti-mouse antibodies coupled to protein A Sepharose resin. The precipitates were analyzed by Western blot using the indicated antibodies.
